# Organ system network analysis and biological stability in critically ill patients

**DOI:** 10.1186/s13054-019-2376-y

**Published:** 2019-03-12

**Authors:** Toshifumi Asada, Kent Doi, Ryota Inokuchi, Naoki Hayase, Miyuki Yamamoto, Naoto Morimura

**Affiliations:** Department of Acute Medicine, The University of Tokyo Hospital, 7-3-1 Hongo, Bunkyo, Tokyo 113-8655 Japan

**Keywords:** Principal component analysis, Organ dysfunction, Organ system network, Homeostasis, Stability

## Abstract

**Background:**

Continuous coordination among organ systems is necessary to maintain biological stability in humans. Organ system network analysis in addition to organ-oriented medicine is expected to improve patient outcomes. However, organ system networks remain beyond clinical application with little evidence for their importance on homeostatic mechanisms. This proof-of-concept study examined the impact of organ system networks on systemic stability in severely ill patients.

**Methods:**

Patients admitted to the intensive care unit of the University of Tokyo Hospital with one representative variable reflecting the condition of each of the respiratory, cardiovascular, renal, hepatic, coagulation, and inflammatory systems were enrolled. Relationships among the condition of individual organ systems, inter-organ connections, and systemic stability were evaluated between non-survivors and survivors whose organ system conditions were matched to those of the non-survivors (matched survivors) as well as between non-survivors and all survivors. We clustered these six organ systems using principal component analysis and compared the dispersion of the principal component scores of each cluster using the Ansari-Bradley test to evaluate systemic stability involving multiple organ systems. Inter-organ connections were evaluated using Spearman’s rank test.

**Results:**

Among a total of 570 enrolled patients, 91 patients died. The principal component analysis yielded the respiratory-renal-inflammatory and cardiovascular-hepatic-coagulation system clusters. In the respiratory-renal-inflammatory cluster, organ systems were connected in both the survivors and the non-survivors. The principal component scores of the respiratory-renal-inflammatory cluster were dispersed similarly (stable cluster) in the non-survivors, the matched survivors, and the total survivors irrespective of the severity of individual organ system dysfunction. Conversely, in the cardiovascular-hepatic-coagulation cluster, organ systems were connected only in the survivors, and the principal component scores of the cluster were significantly dispersed (unstable cluster) in the non-survivors compared to the total survivors (*P* = 0.002) and the matched survivors (*P* = 0.004).

**Conclusions:**

This study demonstrated that systemic instability was closely associated with network disruption among organ systems irrespective of their dysfunction severity. Organ system network analysis is necessary to improve outcomes in severely ill patients.

**Electronic supplementary material:**

The online version of this article (10.1186/s13054-019-2376-y) contains supplementary material, which is available to authorized users.

## Background

The human body comprises diverse organ systems with specific functions that continuously coordinate with each other to optimize their functions to maintain biological stability [1–3]. The negative outcomes of individual organ system dysfunctions are well known [4, 5]. However, disruptions in organ system interactions have been increasingly recognized to be associated with morbidity and mortality [6]. Incorporation of organ system networks with conventional medical approaches that focus on individual organ systems is expected to further advance medicine [7]. However, the field of network medicine is in an early phase, and the roles of organ system networks in the maintenance of biological stability remain unclear [2].

An organ system is a biological unit in the nested structure of the human body. Each system contains a hierarchical structure of tissues, cells, and molecules [7–9]. Studies indicate that higher-level biological stability depends on the balance of functions of and interactions among the components of the lower levels of this hierarchical structure [8]. For example, cellular homeostasis is predominantly determined by both the functionality and integrity of molecules such as enzymes within the cell [10–12]. Individual functions of the constituent cells and intercellular signaling aid in maintaining tissue homeostasis [10, 13, 14]. In case of tissue dysfunction within one organ system, the surrounding tissues can compensate appropriately to maintain organ functionality [15–17]. However, whether this analogous relationship is preserved within the higher levels of the hierarchical structure remains unclear, and little evidence exists regarding an association between systemic stability involving several organ systems and balance of individual organ system conditions and inter-organ networks.

A strategy to evaluate the organ system networks is necessary as the first step in their application to clinical settings. However, the dynamic, multidimensional, and non-linear aspects of organ functions and their interactions challenge the assessment of upstream organ system networks by evaluation of constituent organs [18]. Thus, we focused on the concept that systemic stability arises as a result of organ system coordination [8, 10]. Our previous report using network analysis showed a more disrupted organ system network in non-survivors compared with survivors, which was independent of individual organ system dysfunctions [19]. The study indicated the impact of networks among organ systems on the ultimate whole-body stability, i.e., life. If organ system networks are closely associated with systemic stability, a novel strategy can be adopted utilizing systemic stability evaluation to assess downstream organ system networks, which can accelerate organ system network analysis. In the current study, we examined the relationship between systemic stability and organ system networks using a different approach, principal component analysis.

## Methods

### Study outline

The purpose of the study was to examine the relationship among systemic stability, individual organ system conditions, and connections among organ systems involved in the mechanism of stability. A closed model was built to first determine specific organ systems to be examined. Principal component analysis was performed to detect organ system clusters comprising the selected organ systems. In each cluster, the conditions and the network of constituent organ systems, and the stability of the cluster were evaluated using clinical variables and principal component scores (Additional file 1: Figure S1).

### Patient enrollment and data collection

This was a prospective observational study. Patients aged ≥ 18 years who were admitted to the intensive care unit (ICU) of the University of Tokyo Hospital were eligible. Patients admitted after cardiopulmonary resuscitation and those who were monitored without arterial catheter insertion were excluded.

The following clinical variables obtained from the medical records were used in the analyses: age, sex, primary cause for ICU admission, presence of sepsis diagnosed based on the International SCCM/ESICM/ACCP/ATS/SIS definition [20], presence of surgery, presence of hypotension requiring vasopressors, acute physiology and chronic health evaluation (APACHE) II score [21], and Sequential Organ Failure Assessment (SOFA) score [5]. Blood samples were collected from arterial catheters and processed immediately upon ICU admission. The following variables reflecting organ system conditions were measured for each patient: ratio of partial arterial oxygen pressure to fraction of inspired oxygen for the respiratory system [4, 5], blood lactate concentration for the cardiovascular system [22–24], plasma neutrophil gelatinase-associated lipocalin (NGAL) level for the renal system [25], serum bilirubin level for the hepatic system [4, 5]; platelet count for the coagulation system [4, 5], and C-reactive protein for the inflammatory system [26, 27]. Plasma NGAL levels were measured using a commercially available Triage NGAL device (Alere Medical, San Diego, CA). All other measurements were conducted at the University of Tokyo Hospital Clinical Laboratory.

### Categorization of enrolled patients

Patients who died during their hospital stay were defined as non-survivors, whereas those who were discharged from the hospital alive were defined as survivors. The Matching R package was used to select the group of survivors whose organ system conditions at ICU admission matched those of the non-survivors at the same time (i.e., matched survivors). This matching is assumed to minimize the potential impact of differences in organ system conditions on the evaluation of the impact of the networks on systemic stability. For matching, propensity score matching was used. Briefly, first, a prediction model was obtained by multivariable logistic regression analysis with death event as the outcome variable and six representative variables of organ systems as independent variables. Each patient’s probability of mortality was calculated using the prediction model, and the probability for optimal matching was utilized [28].

### Assessment of multiple organ system clusters and inter-organ connectedness

The condition of individual organ systems was evaluated using their representative variables. Principal component analysis was conducted to detect organ system clusters and quantitatively evaluate their stability. Principal component analysis is a multivariate analysis to integrate *N* correlated variables with *N* uncorrelated principal components without changing their information. Principal components are obtained by maximizing the variance of principal component scores, which are calculated by summing up the product of original values of the variables and their eigenvectors. The eigenvalues represent the amount of information of each component, which is the maximum in the first principal component, followed by the second principal component. Using this analysis, the six organ systems were integrated by considering the first and second principal components as clusters comprising multiple organ systems. In the current study, principal component scores were approximated with the exclusion of organ systems whose absolute value of component eigenvector was < 0.1 from the calculation (Additional file 2: Table S1). The principal component scores were used to evaluate the degree of incompetency of multiple organ system clusters. The scores were also used to evaluate the stability of the clusters. As clinical indicators of homeostatic state, such as blood pH, are tightly regulated within a limited range around a specific value [8, 10], dispersion of the principal component scores was evaluated as indicators of organ system cluster stability in two independent approaches. First, the statistical “variability” of the principal component scores was compared among the groups using the Ansari-Bradley test [29]. Second, the degree of “deviation” from a reference value (with 0 set as the average of scores of all enrolled patients) of principal component scores was compared in individual patients.

The connection among organ systems in each cluster was evaluated using statistical correlation in two approaches. First, multiple linear regression analysis was conducted with one representative variable set as the dependent variable and the other variables in the same cluster set as independent variables. Second, Spearman’s rank correlation coefficient was used to assess correlations among the variables. The inter-organ connection was interpreted as preserved when both the partial regression coefficient by multiple linear regression analysis and Spearman’s rank correlation coefficient between two variables were statistically significant.

In summary, the survivors were compared with the non-survivors for the stability of organ system clusters detected by principal component analysis, which was evaluated by dispersion of principal component scores; for constituent organ system conditions, which was evaluated by representative variables; and for the network among the organ systems in each cluster, which was evaluated by statistical correlations among variables.

### Statistical analysis

Continuous variables were described as medians with interquartile ranges (IQRs) and compared using the Wilcoxon rank-sum test. Categorical variables were described as percentages and compared using the chi-square test. Distribution of variables was analyzed by the Shapiro-Wilk test. Variables that were not normally distributed were log-transformed and included in multivariable analyses. Probability values of < 0.05 were considered statistically significant. If needed, Bonferroni correction for multiple comparisons was used in correlation analysis. Differences between groups were considered important when Cohen’s *d* was > 0.20. All analyses were performed using R version 3.1.1 (R Project for Statistical Computing, Vienna, Austria; http://www.R-project.org).

## Results

### Patient characteristics

**Fig. 1:**
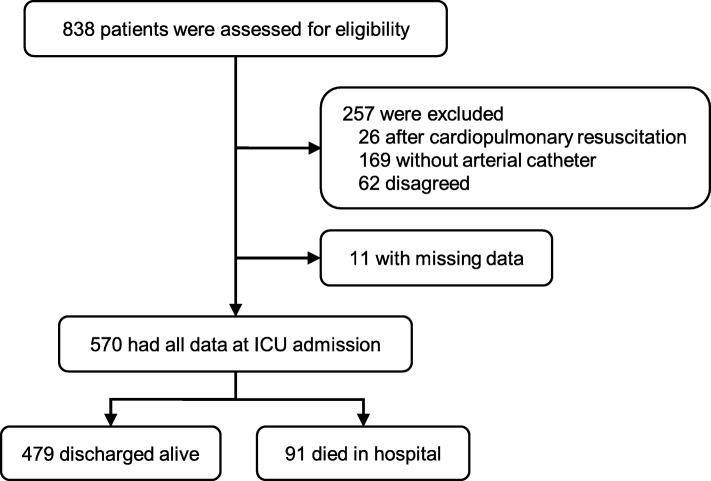
Study flow diagram

A total of 570 patients admitted to our ICU from April 2010 to March 2011, from October 2012 to March 2013, and from September 2014 to March 2015 were included in the study (Fig. 1). During their hospital stay, 91 patients died (the non-survivors), whereas 479 patients were discharged alive from the hospital. The baseline characteristics of the patients are shown in Table 1. Briefly, the baseline conditions of the non-survivors were significantly worse than those of the total survivors according to the SOFA scores and six representative variables. However, we found no important differences in the SOFA scores or the six organ system conditions between the matched survivors and non-survivors based on a Cohen’s *d* of ≤ 0.2.

**Table 1:** Baseline characteristics of the patients

Variable	Non-survivors	Total survivors			Matched survivors		
	(*N* = 91)	(*N* = 479)			(*N* = 91)		
			*P* value^b^	Cohen’s *d*^b^		*P* value^c^	Cohen’s *d*^c^
Age (years)	66 (54–77)^a^	64 (52–73)	0.12	0.21	68 (58–76)	0.88	0.04
Male, no. (%)	59 (65)	295 (62)	0.56		63 (69)	0.53	
Cause for ICU admission, no. (%)			< 0.001			0.20	
Neurological	12 (13)	123 (26)			10 (11)		
Respiratory	16 (18)	22 (5)			7 (8)		
Cardiovascular	11 (12)	63 (13)			16 (18)		
Hepatic	4 (4)	33 (7)			7 (8)		
Renal	7 (8)	11 (2)			2 (2)		
Infection	18 (20)	73 (15)			21 (23)		
Abdominal	7 (8)	53 (11)			12 (13)		
Others	16 (18)	101 (21)			16 (18)		
Sepsis, no. (%)	47 (52)	135 (28)	< 0.001		36 (40)	0.17	
Surgical, no. (%)	10 (11)	206 (43)	< 0.001		30 (33)	< 0.001	
Hypotension, no. (%)	52 (57)	159 (33)	< 0.001		47 (52)	0.55	
APACHE II score	24 (20–30)	17 (13–22)	< 0.001	0.92	19 (14–25)	< 0.001	0.54
SOFA score	9 (6–13)	6 (4–9)	< 0.001	0.82	8 (6–12)	0.12	0.20
PaO_2_/F_I_O_2_	206 (130–331)	324 (217–440)	< 0.001	0.67	214 (134–353)	0.51	0.07
Blood lactate (mmol/L)	2.3 (1.3–4.9)	1.7 (1.0–2.8)	< 0.001	0.55	2.3 (1.2–3.9)	0.44	0.15
Plasma NGAL (ng/mL)	231 (110–518)	114 (60–309)	< 0.001	0.33	188 (84–439)	0.43	0.02
Total bilirubin (mg/dL)	0.9 (0.6–2.0)	0.7 (0.5–1.3)	0.02	0.29	0.9 (0.6–1.8)	0.95	0.19
Platelet count (× 10^4^/μL)	11.0 (4.7–23.1)	18.3 (11.9–24.5)	< 0.001	0.50	14.1 (8.2–18.8)	0.60	0.06
C-reactive protein (mg/dL)	6.4 (0.9–18.5)	1.6 (0.3–8.9)	< 0.001	0.47	3.2 (0.6–12.6)	0.15	0.19

^a^Values denote the number of patients (percentage) or median (interquartile range)

^b^Comparing total survivors to non-survivors

^c^Comparing matched survivors to non-survivors

### Clustering of organ systems

**Fig. 2:**
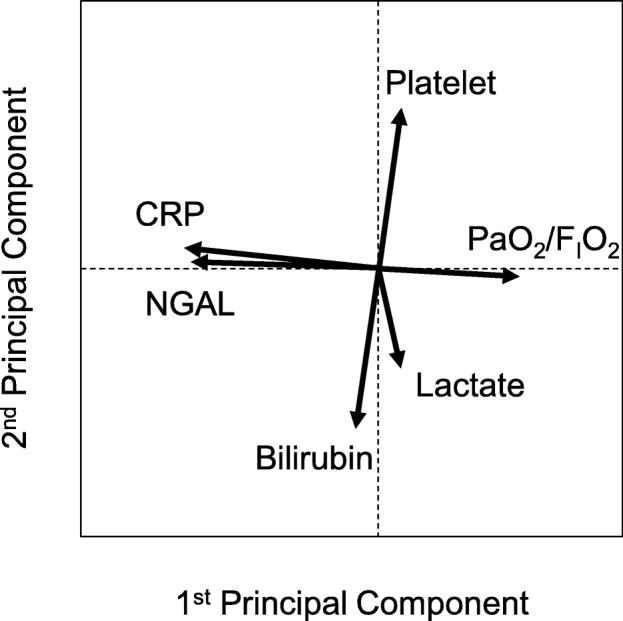
Clustering of the organ systems. Principal component analysis showing that the six organ systems were divided into two clusters: one cluster of the respiratory, renal, and inflammatory systems and one cluster of the cardiovascular, hepatic, and coagulation systems PaO_2_/F_I_O_2_, the ratio of partial pressure arterial oxygen to the fraction of inspired oxygen; NGAL, neutrophil gelatinase-associated lipocalin; CRP, C-reactive protein

Principal component analysis was conducted using the data of all 570 patients to examine clustering of the respiratory, cardiovascular, renal, hepatic, coagulation, and inflammatory systems. Two multiple organ system clusters comprising three organ systems each were detected (Fig. 2): one with the indicators of the respiratory, renal, and inflammatory systems and the other with indicators of the cardiovascular, hepatic, and coagulation systems.

### Evaluation of biological stability by principal component score distribution

**Fig. 3:**
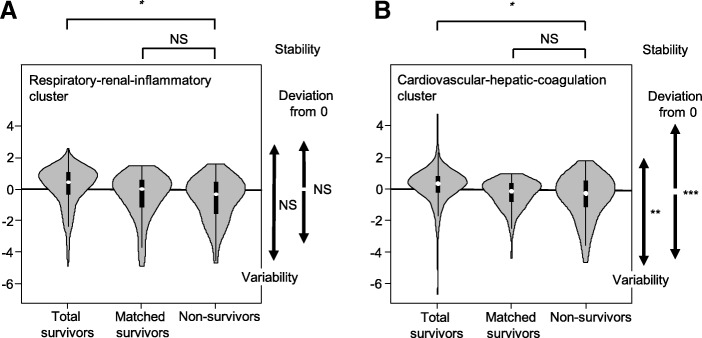
Stability of multiple organ system clusters evaluated by dispersion of principal component scores. White circles indicate medians, and black boxes indicate interquartile ranges of principal component scores for **a** the respiratory-renal-inflammatory cluster and **b** the cardiovascular-hepatic-coagulation cluster. Kernel density estimations are shown on the side of the boxes (gray area). Dispersion of the scores for the respiratory-renal-inflammatory cluster in the non-survivors is not significantly different than those for the total survivors and the matched survivors. However, the dispersion of the scores for the cardiovascular-hepatic-coagulation cluster in the non-survivors is significantly different than that for the survivors. NS, not significant with a Cohen’s *d* ≤ 0.2 (not available for the Ansari-Bradley test). *Difference in medians of principal component scores with a *P* < 0.001. **Statistical variability in the non-survivors evaluated by the Ansari-Bradley test compared to the total survivors (*P* = 0.002) and the matched survivors (*P* = 0.004). ***Deviation from 0 in the non-survivors compared to the total survivors (*P* = 0.003) and the matched survivors (*P* = 0.003)

In our analysis, higher principal component scores indicated better conditions of organ system clusters (Additional file 2: Table S1 provides the eigenvector of each principal component shown in Fig. 2). The principal component score of the respiratory-renal-inflammatory cluster of the non-survivors (− 0.32; IQR, − 1.65 to 0.46) was lower than that of the total survivors (0.45; IQR, − 0.37 to 1.04; *P* < 0.001; Cohen’s *d* = 0.61) but was not importantly different than that of the matched survivors (− 0.04; IQR, − 1.27 to 0.55), with a Cohen’s *d* of 0.13 (Fig. 3a). Similarly, the principal component score of the cardiovascular-hepatic-coagulation cluster of the non-survivors (− 0.30; IQR, − 1.27 to 0.39) was lower than that of the total survivors (0.25; IQR, − 0.30 to 0.71; *P* < 0.001; Cohen’s *d* = 0.64) but was not importantly different than that of the matched survivors (− 0.21; IQR − 0.91 to 0.25), with a Cohen’s *d* of 0.16 (Fig. 3b). These results showed that the principal component scores reflected baseline conditions of the organ systems in all groups.

Conversely, analysis of the statistical variability of the principal component scores by the Ansari-Bradley test showed no significant differences between the total survivors and the non-survivors or between the matched survivors and the non-survivors within the respiratory-renal-inflammatory cluster (*P* = 0.87 and *P* = 0.66, respectively) (Fig. 3a). Conversely, the principal component scores of the cardiovascular-hepatic-coagulation cluster in the non-survivors were significantly dispersed compared to those in the total survivors (*P* = 0.002) and the matched survivors (*P* = 0.004) (Fig. 3b).

Regarding the respiratory-renal-inflammatory cluster, the deviation of the principal component score from the reference value of 0 was 0.77 (IQR, 0.32–1.65) for the non-survivors, which was similar to those for the total survivors (0.91; IQR, 0.42–1.37; *P* = 0.91; Cohen’s *d* = 0.14) and the matched survivors (0.85; IQR, 0.35–1.27; *P* = 0.64; Cohen’s *d* = 0.04). Conversely, for the cardiovascular-hepatic-coagulation cluster, the deviation of the principal component score for the non-survivors (0.78; IQR, 0.36–1.37) was significantly higher than those for the total survivors (0.57; IQR, 0.27–1.02; *P* = 0.003; Cohen’s *d* = 0.43) and the matched survivors (0.42; IQR, 0.25–0.91; *P* = 0.003; Cohen’s *d* = 0.45). These results showed that the dispersion of the scores was not associated with the baseline organ system conditions.

### Stability and isolation in the organ system network

**Fig. 4:**
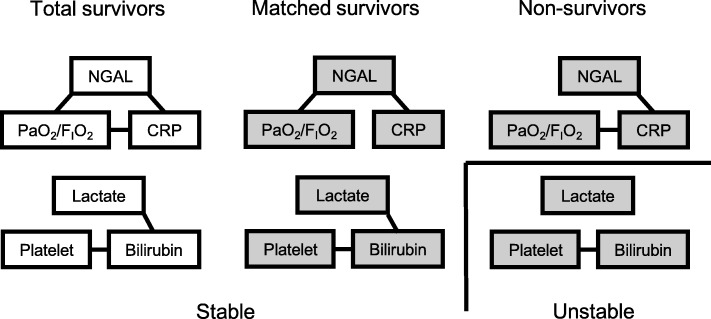
Relationships among organ system dysfunction, networks, and biological stability. Each variable represents one organ system. The variables are connected with each other by lines when the correlations are statistically significant. Organ system dysfunctions in the non-survivors and the matched survivors are worse than that in the total survivors (colored with gray). When the organ systems are networked without isolation, the multiple organ system clusters are stable with narrowly regulated principal component scores. PaO_2_/F_I_O_2_, the ratio of partial pressure arterial oxygen to the fraction of inspired oxygen; NGAL, neutrophil gelatinase-associated lipocalin; CRP, C-reactive protein

Using correlation and multiple linear regression analyses, we further examined the connection among the constituent organ systems in each cluster (Fig. 4 and Additional file 2: Tables S2–S7). We found that the respiratory, renal, and inflammatory systems were connected without isolation in all groups. We also found that the cardiovascular, hepatic, and coagulation systems were connected in the survivors. However, in the non-survivors, the cardiovascular system, represented by blood lactate, was isolated from the other two organ systems.

In the non-survivors, the organ system cluster was unstable, indicated by more dispersed principal component scores than that in the survivors, with isolation of the cardiovascular system in the cardiovascular-hepatic-coagulation cluster. In contrast, the respiratory-renal-inflammatory cluster was interpreted as stable both in the survivors and the non-survivors based on the similarly dispersed principal component scores among the groups irrespective of individual organ system dysfunctions. Therefore, principal component analysis revealed the close relationship between the organ system network and the systemic stability.

## Discussion

Organ system network analysis is a new approach to understand the complexity of life-threatening events, including sepsis and aging [2, 6, 8, 30]. Using a closed model of certain organ systems and principal component analysis, we identified two organ system clusters, respiratory-renal-inflammatory and cardiovascular-hepatic-coagulation, in ICU patients (Fig. 2). Our results showed the concurrence of organ cluster instability indicated by more dispersed principal component scores and cardiovascular system isolation in the non-survivors, whereas the stability of clusters was preserved among groups with similar score dispersions despite severe dysfunction when the constituent organ systems were networked (Fig. 4). This parallel relationship between systemic stability and organ system network agrees with previous reports suggesting that systemic stability arises as the result of appropriate organ system networks [8, 10]. Our observations provide additional evidence for the impact of organ system networks on systemic stability and indicate the potential utility of systemic stability evaluation to promote organ system network analysis.

In the present study, we chose one representative variable for each organ system. Two aspects regarding variable selection should be addressed. First, the six selected variables in the current study are limited in reflecting individual organ systems, and not all variables including the above are specific to individual organ systems. Second, coordination of multiple organ systems has been demonstrated to participate in the maintenance of biological stability by basic and clinical studies; therefore, many other variables should ideally be included to evaluate organ networks. In addition, longitudinal data are preferable to evaluate dynamic changes in organ function and organ networks, as evidenced by a previous study indicating that longitudinal evaluation of the SOFA scores was useful for predicting outcomes in critically ill patients [31]. Because multiple variables with serial analysis will certainly provide a more precise view of the dynamic and multidimensional organ networks, comprehensive big data analysis with more variables and longitudinal data is required in the future.

Principal component analysis allowed the quantitative evaluation of the integrative condition of organ system clusters using principal component scores. Similar to other existing homeostatic variables, the scores were used as indicators of biological stability by their dispersion. To the best of our knowledge, this was the first attempt to evaluate the stability of multiple organ system clusters using principal component scores. In cases where the principal component scores in each cluster were distributed similarly among groups, the organ cluster stability was considered to be maintained. As a similar dispersion was observed simultaneously across the networked organ systems, the regulated principal component scores were interpreted as the result of homeostatic mechanisms aimed at maintaining the stability of organ system clusters in response to the dysfunction of the constituent organ systems. Actually, in the current study, although the principal component scores were higher in the survivors than the non-survivors, the dispersion of scores were similar in the respiratory-renal-inflammatory cluster. We interpreted this result as an appropriate response of the organ network to maintain stability in response to organ system dysfunctions. This interpretation is supported by the “allostasis” concept, an adaptive process of reestablishing homeostasis to maintain stability of the organism [3].

It is undisputable that severe dysfunction in individual organ systems causes biological instability, ultimately leading to death. During the natural course of diseases, organ system dysfunction is compensated by other organ systems. Biological instability can sometimes occur when the compensative mechanisms are disrupted. Conventional medicine has focused on organ failure and biological instability, which put the emphasis on organ support as therapy and substitutional effort to fix organ dysfunction and the homeostatic parameters. However, this strategy remains insufficient to fight against life-threatening illnesses. Indeed, there is strong evidence that permitting hypercapnia with low pH to avoid excessive ventilation might be beneficial in acute respiratory distress syndrome [32] and that strict glucose control is associated with high mortality in ICU patients [33]. Based on the close relationship between organ system networks and systemic stability, we emphasize the necessity of organ system network evaluation to improve outcomes of critically ill patients. To promote organ system network analysis, comprehensive as well as organ-oriented analyses are necessary regarding systemic stability as the result of appropriate organ system networks.

The present study has several limitations. First, the study evaluated only six variables measured at the time of ICU admission. More variables with serial sampling will be preferable to generalize the conclusions by considering dynamic and multidimensional aspects of organ system conditions and their networks. Second, the organ system networks were analyzed by population correlation, and dispersion of the principal component scores was compared among the groups as well. Further investigation will be required to clarify whether the same association between systemic stability and the network is observed in individuals. Third, as the correlations among the organ systems were determined dichotomously with *P* values, the quality of the networks was not evaluated. The results suggested that strong, statistically detectable organ system networks are required to compensate and maintain stability. Fourth, although principal component analysis divided the selected organ systems into two clusters, there is a possibility that the organ systems in one cluster might influence the organs in the other cluster because their eigenvectors of the components were not zero (see Additional file 2: Table S1). Fifth, although the conditions of organ systems were matched, not all the parameters at baseline were adequately matched among the groups because of the disease severity in the non-survivors. The APACHE II scores were higher in the non-survivors than in the matched survivors. Although the degree of organ system dysfunction, organ system networks, and stability of the clusters were calculated and evaluated by matched variables in the closed model of selected organ systems, differences in other organ system conditions might influence the results. However, based on the results, we interpreted that the significantly worse APACHE scores in the non-survivors were due to systemic instability, with organ system network disruption represented by worse homeostatic variables such as the blood pH. Finally, this was a single-center study, and a multicenter study is preferable to reduce the impact of different medical management approaches on the study variables.

## Conclusions

This is the first study to provide quantitative evidence for the significance of organ system networks in biological stability. The necessity of organ system network evaluation to improve patient outcomes is emphasized, and comprehensive analysis is necessary regarding systemic stability as the result of appropriate organ system networks to promote organ system network analysis.

## Additional files

Additional file 1: **Figure S1.** Schematic view of methodology. (PDF 79 kb)

Additional file 2: **Table S1.** Eigenvectors of each principal component. **Table S2.** Correlations among respiratory, renal and inflammatory systems in total survivors. **Table S3.** Correlations among cardiovascular, hepatic and coagulation systems in total survivors. **Table S4.** Correlations among respiratory, renal and inflammatory systems in matched survivors. **Table S5.** Correlations among cardiovascular, hepatic and coagulation systems in matched survivors. **Table S6.** Correlations among respiratory, renal and inflammatory systems in non-survivors. **Table S7.** Correlations among cardiovascular, hepatic and coagulation systems in non-survivors. (DOCX 22 kb)

## Abbreviations

APACHE: Acute physiology and chronic health evaluation
ICU: Intensive care unit
IQR: Interquartile range
NGAL: Neutrophil gelatinase-associated lipocalin
SOFA: Sequential Organ Failure Assessment
