# Supplementary figures and images for: Organ system network analysis and biological stability in critically ill patients
Source: Crit Care. 2019 Mar 12;23:83. doi: 10.1186/s13054-019-2376-y (PMC6417231; doi:10.1186/s13054-019-2376-y)

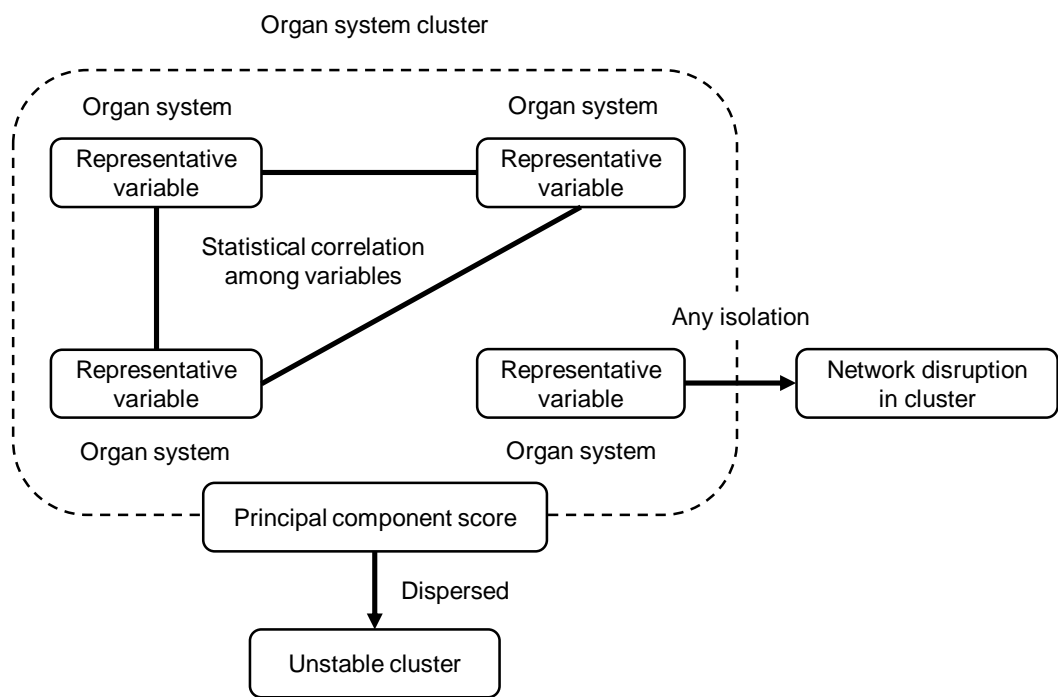

Supplemental Figure 1. Schematic view of methodology

Supplement: Supplementary file 1 — Figure S1. Schematic view of methodology. (PDF 79 kb) [file 13054_2019_2376_MOESM1_ESM.pdf]
