# Supplementary material for: Organ system network analysis and biological stability in critically ill patients
Source: Crit Care. 2019 Mar 12;23:83. doi: 10.1186/s13054-019-2376-y (PMC6417231; doi:10.1186/s13054-019-2376-y)
Supplement: Supplementary file 2 — Table S1. Eigenvectors of each principal component. Table S2. Correlations among respiratory, renal and inflammatory systems in total survivors. Table S3. Correlations among cardiovascular, hepatic and coagulation systems in total survivors. Table S4. Correlations among respiratory, renal and inflammatory systems in matched survivors. Table S5. Correlations among cardiovascular, hepatic and coagulation systems in matched survivors. Table S6. Correlations among respiratory, renal and inflammatory systems in non-survivors. Table S7. Correlations among cardiovascular, hepatic and coagulation systems in non-survivors. (DOCX 22 kb) [file 13054_2019_2376_MOESM2_ESM.docx]

**Table S1. Eigenvectors of each principal component**

| Variable | 1^st^ Principal Component | 2^nd^ Principal Component |
| --- | --- | --- |
| PaO_2_/F_I_O_2_ | 0.47736915*^a^* | –0.03236429 |
| NGAL | –0.60429977 | 0.01048927 |
| CRP | –0.62410438 | 0.06696707 |
| Lactate | 0.07943143 | –0.40440938 |
| Bilirubin | –0.06756784 | –0.64763819 |
| Platelet count | 0.08099043 | 0.64138582 |
| Eigenvalue | 1.8402 | 1.3327 |
| % of Variance | 30.7 | 22.2 |

*^a^* If absolute value of eigenvector was less than 0.1, the variable was excluded from the calculation of principal component score.

**Table S2.** **Correlations among respiratory, renal and inflammatory systems in total survivors**

| Variable | PaO_2_/F_I_O_2_ | NGAL | CRP |
| --- | --- | --- | --- |
| PaO_2_/F_I_O_2_ |  | –0.4041*^a^* | –0.3017*^a^* |
| NGAL |  |  | 0.5855*^a^* |
| CRP |  |  |  |

*^a^* Values of Spearman rank correlation coefficient were significant with and without Bonferroni correction.

**Table S3.** **Correlations among cardiovascular, hepatic and coagulation systems in total survivors**

| Variable | Lactate | Bilirubin | Platelet |
| --- | --- | --- | --- |
| Lactate |  | 0.2124*^a^* | –0.1558*^a^* |
| Bilirubin |  |  | –0.4065*^a^* |
| Platelet |  |  |  |

*^a^* Values of Spearman rank correlation coefficient were significant with and without Bonferroni correction.

**Table S4.** **Correlations among respiratory, renal and inflammatory systems in matched survivors**

| Variable | PaO_2_/F_I_O_2_ | NGAL | CRP |
| --- | --- | --- | --- |
| PaO_2_/F_I_O_2_ |  | –0.3676*^a^* | –0.3301*^a^* |
| NGAL |  |  | 0.5860*^a^* |
| CRP |  |  |  |

*^a^* Values of Spearman rank correlation coefficient were significant with and without Bonferroni correction.

**Table S5.** **Correlations among cardiovascular, hepatic and coagulation systems in matched survivors**

| Variable | Lactate | Bilirubin | Platelet |
| --- | --- | --- | --- |
| Lactate |  | 0.3109*^a^* | –0.2071*^b^* |
| Bilirubin |  |  | –0.3704*^a^* |
| Platelet |  |  |  |

*^a^* Values of Spearman rank correlation coefficient were significant with and without Bonferroni correction.

*^b^* A value of Spearman rank correlation coefficient was significant without Bonferroni correction.

**Table S6.** **Correlations among respiratory, renal and inflammatory systems in non-survivors**

| Variable | PaO_2_/F_I_O_2_ | NGAL | CRP |
| --- | --- | --- | --- |
| PaO_2_/F_I_O_2_ |  | –0.2585*^a^* | –0.4207*^b^* |
| NGAL |  |  | 0.4841*^b^* |
| CRP |  |  |  |

*^a^* A value of Spearman rank correlation coefficient was significant without Bonferroni correction.

*^b^* Values of Spearman rank correlation coefficient were significant with and without Bonferroni correction.

**Table S7.** **Correlations among cardiovascular, hepatic and coagulation systems in non-survivors**

| Variable | Lactate | Bilirubin | Platelet |
| --- | --- | --- | --- |
| Lactate |  | 0.0959 | –0.0534 |
| Bilirubin |  |  | –0.5169*^a^* |
| Platelet |  |  |  |

*^a^* A value of Spearman rank correlation coefficient was significant with and without Bonferroni correction.
